# Type I and II Interferons in the Anti-Tumor Immune Response

**DOI:** 10.3390/cancers13051037

**Published:** 2021-03-02

**Authors:** Sarah E. Fenton, Diana Saleiro, Leonidas C. Platanias

**Affiliations:** 1 Robert H. Lurie Comprehensive Cancer Center of Northwestern University, Chicago, IL 60611, USA; sarah.fenton@northwestern.edu (S.E.F.); diana.saleiro@northwestern.edu (D.S.); 2 Division of Hematology-Oncology, Department of Medicine, Feinberg School of Medicine, Northwestern University, Chicago, IL 60611, USA; 3 Department of Medicine, Jesse Brown Veterans Affairs Medical Center, Chicago, IL 60612, USA

**Keywords:** interferons, cancer, dendritic cell, macrophage, T cell, cancer immunoediting

## Abstract

**Simple Summary:**

Interferons are cytokines that play key roles in the activation of cellular components of the immune response, such as dendritic cells, macrophages and T cells. Generally, these cytokines promote anti-tumor immune responses, but under some circumstances, prolonged exposure to them can lead to suppression of the immune response. This review focuses on the immunostimulatory versus immunosuppressive roles of interferons and the mechanisms mediating such effects on both malignant cells and cells of the immune system.

**Abstract:**

The interferons (IFNs) are essential components of the immune response against infections and malignancies. IFNs are potent promoters of the anti-tumor response, but there is also evidence that feedback mechanisms regulated by IFNs negatively control immune responses to avoid hyper-activation and limit inflammation. This balance of responses plays an important role in cancer surveillance, immunoediting and response to anticancer therapeutic approaches. Here we review the roles of both type I and type II IFNs on the control of the immune response against malignancies in the context of effects on both malignant cells and cells of the immune system in the tumor microenvironment.

## 1. Introduction

The intersections between malignancy and the immune system and the communications between malignant cells and cells of the immune system have emerged as central to understanding oncogenesis and tumor progression [1]. With the introduction of immunomodulatory anticancer therapies such as antibodies against PD-L1 and CTLA-4, as well as an increased understanding of the importance of immune activation in the response to chemotherapeutic agents, research into immunity within the tumor microenvironment (TME) has continued to evolve [2]. Central to this progress has been the hypothesis of cancer immunoediting. This model posits that the immune system initially defends the host against cancer development [3]. However, some malignant cells can survive this protective immune response, resulting in their escape and continued tumor growth [4,5]. Thus, immune pressure on malignant cells can initially induce tumor cell death, but later allow tumor growth by reducing the ability of the host to stimulate a protective immune response in favor of a quiescent TME. Signaling by the interferon (IFN) receptors has been shown to be central to both mechanisms of immune surveillance and escape [4,5]. 

The IFNs evolved to counter viral and bacterial insults by activating the adaptive immune response and reducing the propagation of infection [6]. IFNs also activate negative feedback signaling mechanisms that aim to prevent excess IFN-mediated inflammation and tissue damage [7,8]. This dual role for IFN signaling is critical for the optimal regulation of responses to infection and antineoplastic effects, but it is also coopted by malignant cells to allow ongoing survival and growth [4]. Understanding the mechanisms of actions of IFNs and how they alter the function of specific cell populations within the TME is important for the development of novel immune therapies. Here we discuss the role of type I and type II IFNs at the level of individual immune cell populations and malignant cells and review the mechanisms by which IFNs regulate anti-tumor immune responses. 

## 2. Interferon Production and Signaling 

IFNs are released predominantly by immune and stromal cells to effect a multitude of cellular responses [9,10]. These cytokines are divided into three classes, type I, type II and type III IFNs, and utilize the type I, II and III IFN receptors, respectively [11]. Upon target cell receptor binding, IFNs induce the expression of unique genes, called IFN-stimulated genes (ISGs), which ultimately mediate IFN-dependent biological effects [12,13]. As not much is known yet about the roles of type III IFNs in the TME, this review will focus on type I and type II IFNs.

### 2.1. Type I Interferons

Type I IFNs (IFNIs) are the largest subgroup of IFNs, and include interferon α (IFNα), interferon β (IFNβ), interferon ε, interferon κ and interferon ω in humans, as well as interferon δ and interferon τ in other mammals [14,15,16]. IFNIs signal via the type I IFN receptor (IFNAR) that is expressed on nearly every cell type and is composed of the receptor subunits IFNAR1 and IFNAR2 [17,18]. In malignant cells, nuclear leakage of DNA and other damage-associated molecular patterns (DAMPs) into the cytosol can be sensed by pattern recognition receptors (PRRs) such as retinoic acid-inducible gene I (RIG-I), DNA-Dependent Activator of IFN-Regulatory Factors and IFI16 to stimulate IFNI production [18]. DAMPs released into the TME interact with endosomal Toll-like receptors (TLRs) in immune cells to activate IFNI production [19]. Additionally, in the cytoplasmic compartment of immune cells, tumor DNA binds cyclic GMP-AMP synthase (cGAS) and acts in conjunction with Stimulator of Interferon Genes (STING) signaling to initiate IFNI production [20,21,22]. IFNI binds to its receptor at the cell surface, activating several signaling pathways. These include the Janus Kinase/Signal Transducer and Activator of Transcription (JAK/STAT) pathways, where complexes of activated STAT proteins translocate to the nucleus and initiate transcription of ISGs by binding to specific promoter interferon-sensitive response elements (ISREs) and IFNγ-activated site (GAS) elements [23]. Other activated pathways include those associated with mitogen-activated protein kinases (MAPKs) (particularly the p38 MAPK signaling cascade) and phosphoinositide 3-kinase (PI3K)/mTOR cascades [9,10,24]. 

### 2.2. Type II Interferon 

The type II IFN class (IFNII) is composed of only one member, IFN gamma (IFNγ) [10]. IFNγ is mainly produced by natural killer cells (NKs), CD8+ T cells, CD4+ T helper type 1 (Th1) cells and by a specific subtype of macrophages and dendritic cells (DCs) [7]. IFNγ binds to the type II IFN receptor (IFNGR) which is composed of two subunits, IFNGR1 and IFNGR2 [25]. Following IFNGR dimerization, the JAK/STAT pathway is activated, altering gene expression through activation and binding of different STAT1 and/or STAT3 complexes to GAS elements in the promoter region of ISGs [10]. IFNII signaling also activates non-STAT pathways that play important roles in eliciting biological responses, including MAPK, PI3K, JNK, CamKII and NF-κB [26]. 

## 3. Effects of Interferons on the Tumor Microenvironment 

### 3.1. Dendritic Cells 

Dendritic cells (DCs) present antigens and produce cytokines to activate other immune cell populations [27]. In the TME, contact with tumor derived DNA and other DAMPs promotes DC activation and IFNI production through the STING/IFN-regulatory factor (IRF) 3 and/or TLR/IRF pathways [17,22]. Exposure to IFNI is then required for full maturation of DCs, characterized by the expression of the costimulatory molecules CD40, CD80 and CD86 and major histocompatibility complex (MHC) class II molecules [28,29]. Additionally, IFNI exposure improves the ability of DCs to present tumor antigens on their cell surface [30,31,32,33] and promotes DC migratory capabilities [34]. Thus, IFNI also increases the ability of DCs to recruit and activate CD4+ Th1 and CD8+ T cells to the TME [31,35]. Additionally, DCs exposed to IFNI produce high levels of interleukin 12 (IL-12) and IL-15, stimulating further downstream immune activation [36,37].

Plasmacytoid dendritic cells (pDCs) are a specialized subset of DCs that release high levels of IFNI in response to foreign antigens and link innate and adaptive immunity [38]. Because pDCs are capable of rapidly secreting much higher levels of IFNI than conventional DCs due to constitutive expression of IRF7, they are thought to play a critical role in the initial immune response against tumor cells [29,39]. In an orthotopic in vivo mammary tumor model, administration of TLR7 ligand was shown to activate tumor-associated pDCs, inducing tumor regression. Neutralization of IFNI activity prevented this response [40]. However, some studies of patient tumors have found that pDC tumor infiltration correlates with poor outcomes [41]. This may be due to pDC co-localization with regulatory T cells (Tregs) in the TME, which decreases responsiveness of tumor infiltrating pDCs, reducing IFNα production [29,42].

Similar to IFNI, IFNγ acts on DCs inducing the expression of cytokines, costimulatory and MHC molecules and proteins involved in the antigen-presentation process, ultimately leading to activation of other immune cells [43,44,45]. IFNγ-induced STAT1-dependent activation of IRF1 regulates expression of the MHC class I locus, while the IFNγ-induced ISG CIITA upregulates expression of MHC class II genes [45]. IFNγ also changes the composition of the proteasome, altering its function to a specialized immunoproteasome, which increases the amount and variety of peptides available for antigen presentation by MHC class I proteins at the surface of DCs [45]. In an in vivo study, co-injection of IFNγ and TLR ligands resulted in higher DC activation and migration from the site of immunization to the draining lymph nodes and a consequent increase in T cell activity [46].

**Figure 1:**
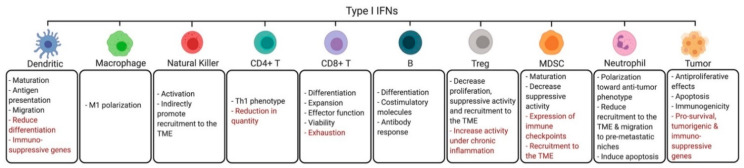
Effects of type I interferons (IFNIs) on malignant cells and cells of the immune system. Exposure to type I IFNs alters the function of target cells within the tumor microenvironment (TME) in ways that promote anti-tumor effects (black) or counter such effects (red).

**Figure 2:**
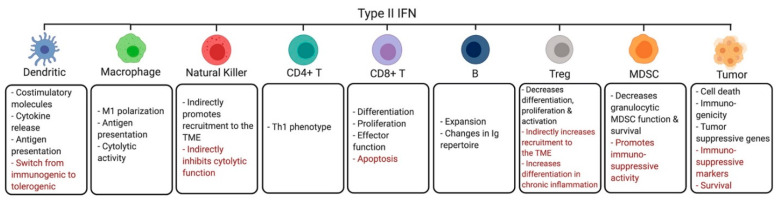
Effects of the type II interferon (IFNII) on malignant cells and different types of immune cells. Exposure to type II IFN alters the function of the tumor and immune cells within the TME in ways that promote anti-tumor effects (black) or counter such effects (red).

Although IFNs promote migration and activation of DCs in the TME initially, at later stages sustained signaling through either the IFNI or IFNII receptors can lead to suppressive effects on DCs. Chronic or prolonged exposure to IFNIs can impede DC differentiation and homeostasis and upregulate immunosuppressive molecules such as PD-L1 on DCs [47,48,49]. Moreover, prolonged exposure to IFNγ induces an IDO-dependent switch of immunogenic DCs to tolerogenic DCs, which favor activation of Tregs [50,51,52]. Notably, GSK-3β inhibitors block IFNγ-mediated IDO expression, enhancing the activity of DC-based vaccines in vivo [53]. Overall, these findings underscore the importance of a better understanding of both IFN-dependent stimulatory and suppressive mechanisms in DCs (Figure 1 and Figure 2) in order to develop novel immunotherapeutic approaches.

Clinically, interest in manufactured DCs and DC-stimulating/based therapies has been growing [54]. Studies in acute myeloid leukemia (AML) models suggest that monocytes reprogrammed with lentiviral vectors to co-express GM-CSF, IFNα2 and antigens differentiate into induced DCs that potently activate anti-tumor T and B cells, providing a potential new therapeutic approach to address minimal residual disease following chemotherapy for AML patients [55]. In pre-clinical breast cancer models, restoration of IFNI activity in the TME via administration of TLR agonists increases activation of DCs and reduces mammary tumor growth [56]. As research into DC-based treatments has continued, IFNα exposure has been identified as an important step to allow vigorous immune responses following treatment and to improve efficacy of anti-tumor vaccines [57,58,59,60]. Finally, several clinical trials are currently underway to study the outcomes of STING agonists alone or in combination therapies [61]. These agonists induce IFNI production, which ultimately activates IFNI signaling in multiple components of the immune system, including DCs [62]. 

### 3.2. Macrophages

Macrophages phagocytize cellular debris to act as antigen presenting cells and produce cytokines such as TNFα and IL-6 in response to foreign materials and exogenous stimuli [63]. Both IFNIs and IFNγ increase macrophage activity by polarizing immature monocytes into a mature anti-tumor M1 phenotype [64,65]. More specifically, IFNγ upregulates expression of MHC molecules at the cell surface of macrophages and controls phagosome functions, increasing the repertoire of antigens available for presentation [45,64,66,67,68,69]. Together, these changes facilitate macrophage-mediated activation of T cells and the adaptive immune response [70]. 

In preclinical breast cancer models, transplantation of engineered TIE2-expressing macrophages that express IFNα reprograms the TME, inducing greater DC activation and CD8+ T cell cytotoxicity against tumor cells [71]. In another study, using a poorly immunogenic breast cancer mouse model, CSF-1R inhibitors were shown to block macrophage immunosuppressive activity by inducing IFNI signaling and expression of ISGs in the TME, synergizing with cisplatin therapy [72]. Novel therapeutic strategies are under development to enhance the anti-tumor functions of macrophages in the TME. One such approach involves blocking the binding between CD47 and signal regulatory protein alpha (SIRPα), increasing tumor cell phagocytosis by macrophages [73,74]. Notably, a recent study demonstrated that the combination of CD47/SIRPα inhibition with activation of CD40 signaling using a SIRPα-Fc-CD40L fusion protein also enhances type I interferon responses in macrophages, increasing effector T cell activity, further blocking tumor growth [75]. In another study, Th1-derived IFNγ was shown to increase macrophage-mediated anti-tumor cytotoxic activity by inducing the expression of proteases, granzyme A/B, and NK cell-related genes (e.g., *NKG2D*) and by promoting CXCL9 and CXCL10 secretion by macrophages [75]. In an ovarian cancer model, GM-CSF and IFNγ produced by T cells expressing the chimeric NKG2D receptor increased activation of macrophages in the TME, IL12p40 production and antigen presentation by macrophages [76]. Additionally, IFNγ was shown to induce macrophages to produce nitric oxide and increase direct lysis of cancer cells [77]. Thus, IFNs sustain an anti-tumor function of macrophages in the TME, driving changes that result in direct and immune-mediated tumor cell death (Figure 1 and Figure 2). 

### 3.3. Natural Killer Cells

In addition to DCs and macrophages, NKs form another critical component of the innate immune response [78,79]. Cytokines such as IFNI, IL-12 and IL-15 drive the activation of NKs [80,81]. Moreover, STING-induced IFNI-mediated expression of the ISGs CXCL10 and CCL5 in tumor and immune cells has also been shown to increase NK accumulation and activation in the TME [82]. Clinical interest in STING agonists has been growing, and the efficacy of these therapies may depend on the promoting effects of IFNI on anti-tumor NK cytotoxicity [82,83]. In a recent study using a preclinical model of triple negative breast cancer, specific deletion of *Ifnar1* expression in NK cells demonstrated a requirement for IFNI in NK cell-mediated tumor cell lysis [84]. Additionally, overexpression of IRF7 in prostate cancer cells inhibits bone metastases through IFNI-induced NK activation [85], while loss of host IFNI receptor signaling in vivo blocks NK-mediated anti-tumor immune responses and results in increased cancer cell metastasis [86,87,88]. Thus, IFNI plays an important role in regulating the induction of NK cytotoxic and anti-tumor activities in the TME [86]. It is remarkable that in mice with chronic viral infections, sustained activation of IFNI signals results in increased immunosurveillance against malignant cells by increasing the cytolytic function of NKs [89]. These studies suggest that NKs play an important role in immune activation and immune-mediated cell death within the TME and IFNIs are required for this response (Figure 1). 

In IFNGR knockout mice less NKs are present in the TME, and this correlates with decreased survival following tumor implantation [90]. These findings were reversible in mice co-injected with recombinant IFNγ protein and malignant cells via up-regulation of CXCR3 ligands in the tumor cells, demonstrating that IFNγ is required for recruitment of NKs to the TME [90]. In contrast, IFNγ was shown to induce PD-L1 expression on tumor cells, reducing their susceptibility to NK cytotoxicity [91]. Thus, depending on the effect of IFNγ exposure on malignant cells, these can be more or less susceptible to NK-cell induced tumor lysis (Figure 2) [90,91,92].

### 3.4. CD4+ Helper T Cells 

CD4+ Th1 cells function by releasing cytokines and modifying the immune response through activation of macrophages and other T cells [93]. Both IFNI and IFNγ drive polarization of CD4+ T cells towards the anti-tumor Th1 phenotype, preventing differentiation into the protumor Th2 phenotype [29,45,94]. IFNγ signals through STAT1 and downstream activation of T-bet, a regulator of the Th1 lineage that upregulates expression of the IL-12 receptor and IFNγ, creating a positive feedback loop [45,95] (Figure 2). On the other hand, in chronic viral infection models, high levels of IFNI have been correlated with reduced numbers of CD4+ T cells [96], suggesting that sustained IFNI exposure could deplete CD4+ T cells in the TME (Figure 1).

### 3.5. CD8+ Cytotoxic T Cells

CD8+ T cells interact with antigen-presenting cells to differentiate into effector CD8+ T cells, defined as cytotoxic T lymphocytes (CTLs) [97]. Upon antigen recognition and costimulation, a third signal (IFNI or IL-12) is necessary for further differentiation of naïve CD8+ T cells [98]. Moreover, IFNI also promotes the expansion, effector function and survival of CTLs [99,100,101,102]. Studies using human colorectal carcinoma cells have shown that tumor tissue and CTLs from colon cancer patients have decreased expression of IFNAR1 compared to normal colon tissue and CTLs from healthy donors [102,103]. Inactivation of IFNAR1 in CTLs was reported to limit their survival within the TME and undermine the efficacy of chimeric antigen receptor (CAR) T cell treatment in colon cancer models, while genetic stabilization of IFNAR1 improved CTL viability and response to both CAR T cell and anti-PD-1 therapy [103]. Additionally, IFNI was shown to activate STAT3 to promote expression of granzyme B in CTLs, enhancing their effector function [104]. It has been shown that resistance to anti-PD-1 therapy can be reversed with intratumoral administration of a TLR9 agonist, which results in IFNI production in the TME and a consequent increase in the number and quality of CD8+ T cells [105]. However, chronic IFNI signaling can ultimately induce an exhausted T cell phenotype through upregulation of the immune checkpoints PD-1, TIM-3 and LAG-3, suppressing the immune response [29,106] (Figure 1).

IFNγ induces the differentiation, activation, proliferation and survival of tumor specific CD8+ T cells, in part through the induction of regulatory genes including survivin and *Ifi202* [107,108]. Following IFNγ exposure, the cytolytic activity of CD8+ T cells is also increased through upregulation of granzymes and IL-2 receptor expression [45]. However, IFNγ released into the TME may induce apoptosis of activated CD8+ T cells that express high levels of IFNGR, limiting immune activity [109] (Figure 2). 

### 3.6. B Cells

IFNI enhances activation of B cells through upregulation of costimulatory molecules, leading to increased B cell responses [110,111]. Additionally, mice with B cells lacking the IFNI receptor present a reduced IFNI-mediated enhancement of the antibody response and isotype switching compared to mice with wild-type B cells [112]. TLR agonists, which induce intratumoral IFNI production, are currently being developed as a potential therapy against distinct malignancies [113,114]. The TLR9 agonist MGN1703 has been shown to promote a potent interferon response in the lymph nodes, increasing differentiation of B cells and activation of pDCs, NKs, and T cells [113].

IFNγ inhibits B cell proliferation at early stages of activation prior to antigen exposure and at later stages, during B cell maturation. However, following primary antigen exposure, IFNγ promotes B cell propagation [45]. Moreover, exposure to CD4+ Th1 cells and IFNγ inhibits B cell class switching to IgE and promotes switching to IgG2, a class of antibodies that mediate antibody-dependent cytotoxicity [45]. Thus, IFNγ plays a complex role on B cell function, depending on the stage of the cell’s differentiation/activation. Interestingly, a CD11a^hi^FcγRIII^hi^ B cell subpopulation was found to produce IFNγ in early stages of the immune response against bacterial or viral infections or in response to TLR ligands, resulting in macrophage activation [115]. These results suggest a potential new role for these specific B cells in the TME. As activated B cells are capable of inducing comparable CTL-produced IFNγ levels and tumor cell death as DCs [116], vaccines composed of B cells are under investigation [117]. For their success, it will be important to consider the effects of both IFNI and IFNγ on these cells (Figure 1 and Figure 2).

### 3.7. Regulatory T Cells

CD4+CD25+FOXP3+ Tregs are a specialized subset of T cells that act to prevent damaging immune overactivity and maintain tolerance to self-antigens by inhibiting the function of effector T cells through the release of inhibitory cytokines, induction of T cell apoptosis and upregulation of immunosuppressive genes [118]. Exposure of Tregs to IFNα in vitro decreases their IL-2-induced proliferation and suppressive activity, ultimately promoting activation of CD4+ Th1 cells [119]. Studies using breast and colon cancer models have shown that IFNα diminishes Treg frequency within the TME [42,120,121]. Specific deletion of IFNAR in Tregs results in increased Treg proliferation and higher expression of PD-1 and CD44 on their cell surface, leading to decreased anti-tumor immune responses and tumor clearing in mouse models of colon cancer and melanoma [122]. Moreover, intratumoral IFNI production via administration of TLR or RIG-I-like receptor ligands inhibits CCL22 expression, a chemokine that serves to attract Tregs to the TME [123]. However, using a T cell-induced colitis model, it has been also shown that under inflammatory conditions IFNI maintains Foxp3 levels and Treg functions [124]. Likewise, under competitive or stress conditions IFNI was shown to sustain Treg development and function [125]. These studies suggest that IFNI could also support Treg immunosuppressive functions under certain conditions [124,125]. Thus, depending on the context, the interaction between IFNIs and Tregs could allow either the induction or suppression of an anti-tumor immune response (Figure 1).

Neuropilin-1 (Nrp1) is a transmembrane receptor that is critical for the function and stability of tumor infiltrating Tregs. *Nrp1*−/− Tregs found within the TME produce IFNγ, which suppress the function of surrounding wild-type Tregs, increase anti-tumor immune activity. Thus, IFNγ released by *Nrp1*−/− Tregs results in increased fragility of other populations of intratumoral Tregs, increasing anti-PD1-driven anti-tumor immune responses [126]. IFNγ also decreases differentiation of T cell precursors into Tregs and induces cell cycle arrest, preventing further proliferation [7,127]. In another study, IFNγ administration reversed SEREX-defined self-Ag-mediated increase in generation/activation of Tregs, resulting in lower tumor incidence and metastasis in vivo [128]. Thus, IFNγ exposure inhibits the differentiation, proliferation, activation and stability of Tregs in the TME. However, excessive IFNγ production by CD4+ Th1 cells, caused by a deficiency in the deubiquitinase USP15, increases the number of Tregs and myeloid derived suppressor cells (MDSCs) in the TME through upregulation of PD-L1 and CXCL12 expression on CD45 negative non-immune cells. This ultimately increases the incidence of methylcholanthrene (MCA)-induced fibrosarcomas [129]. Additionally, in models of chronic inflammation, IFNγ can drive the differentiation of specialized Tregs that inhibit CD4+ Th1 cells [108]. Together, these data suggest that IFNγ directly or indirectly is capable of either stimulating or suppressing Treg function (Figure 2).

Potential therapeutic options including Treg depletion are under study [130]. Interestingly, inhibition of the histone H3K27 methyltransferase enhancer of zeste homolog 2 (EZH2) activity in Tregs induces Treg-mediated pro-inflammatory functions, enhancing IFNγ production by CD8+ and CD4+ Th1 cells and anti-tumor immune responses [131].

### 3.8. Myeloid Derived Suppressor Cells

Tumors are often found to have increased infiltration by MDSCs [132]. MDSCs support an immunosuppressive TME by producing cytokines, such as IL-10, which promote activation of immunosuppressive cells (M2 macrophages and Tregs), by sequestering arginine or cysteine and by producing nitric oxide and reactive oxygen species to block T cell activation [133]. Moreover, IFNs upregulate the expression of the immune checkpoint PD-L1 on the surface of MDSCs [134,135]. Resistance to radiation therapy has been associated with STING-dependent recruitment of MDSCs to the TME [136]. However, other reports have associated IFNI production with reduced MDSC accumulation and activity in the TME [137,138]. Interestingly, treatment with the TLR9 ligand CpG induces IFNI production by pDCs, which results in maturation of MDSCs in vitro [138]. Treatment with either CpG or IFNI in two tumor models resulted in decreased suppressive activity of MDSCs [138]. These data suggest that when IFNIs act directly on MDSCs, they decrease their inhibitory function, restoring the immune system’s ability to eliminate malignant cells. As is often found when studying the anticancer immune response, exposure of MDSCs to IFNI can differentially regulate their response based on several factors, including the presence of other immune cells and inflammatory mediators (Figure 1).

IFNγ produced by CD8+ T cells has been found to stimulate iNOS expression in monocytic MDSC (M-MDSC)-derived macrophages, promoting TLR2 ligand-dependent M-MDSC-induced suppression of T cell activity [139]. In another study, in vitro co-treatment of bone marrow-derived MDSCs with lipopolysaccharide and IFNγ increased MDSC-mediated nitric oxide production and immunosuppressive activity, while blocking development of DCs [140]. Moreover, mast cells enhance the suppressive activity of M-MDSCs through an IFNγ-dependent mechanism [141]. IFNγ promotes production of IL-10 and TGF-β by MDSCs, and these cytokines, in the presence of tumor antigen-stimulated T cells, increase MDSC-induced development of immunosuppressive Tregs [142]. Additionally, prolonged IFNγ signaling drives the expression of immunosuppressive molecules including TGF-β, IL-10, and IDO in MDSCs, leading to reduced T cell activity [143]. However, there is evidence to suggest that IFNγ decreases the survival and function of granulocytic MDSCs [144]. Thus, IFNγ is capable of positively or negatively regulating MDSC suppressive functions (Figure 2).

### 3.9. Neutrophils

Both protumor and anti-tumor roles for neutrophils have been identified and IFNIs play an important part in regulating these functions [145,146,147,148,149]. Andzinski et al. have shown that IFNI therapy, both in mice and humans, promotes polarization of tumor-associated neutrophils (TANs) towards an anti-tumor N1 phenotype [145]. Another recent study demonstrated that β-glucan, an agonist of trained immunity, epigenetically rewires neutrophils into an anti-tumor phenotype in an IFNI signaling-dependent manner [146]. Furthermore, IFNβ downregulates the expression of CXCR2 ligands on the surface of TANs, reducing their recruitment into tumors and inhibiting tumor angiogenesis [147]. IFNβ also induces apoptosis of TANs, limiting their lifespan in the TME [148]. Interestingly, accumulation of neutrophils in distant tissue is thought to mediate the formation of pre-metastatic niches. *IFNb1*−/− mice have increased accumulation of neutrophils in the lungs and a higher rate of metastatic disease, suggesting that IFNI activity reduces metastatic potential through decreasing migration of neutrophils to would-be sites of metastasis [149]. These studies support the hypothesis that IFNIs can support an antitumor immune response by exerting anti-tumor effects on TANs (Figure 1).

### 3.10. γδ T Cells

γδ T cells are a subset of T cells that express a T-cell receptor (TCR) composed of a gamma and delta chain (instead of the α/β TCR that is more commonly expressed in other T cells) and are not MHC restricted [150]. γδ T cells express T-bet and eomesodermin constitutively and so can rapidly differentiate and secrete IFNγ once stimulated with IL-2 and IL-15, promoting anti-tumor immune responses [150]. γδ T cells are also capable of acting as antigen presenting cells and can activate CD8+ and CD4+ Th1 cells [151]. Finally, the majority of γδ T cells can directly kill tumor cells through granzyme and perforin secretion or indirectly through IFNγ and TNF production [151]. Loss of IFNγ production by γδ T cells resulted in increased tumorigenesis following MCA challenge [152]. In an in vivo model where TCRδ deletion abrogated T cell-mediated immune response, the reintroduction of γδ T cells restored anti-tumor immune function. This effect required IFNγ and perforin production by γδ T cells and other lymphocytes [153]. However, the γδ T cells found in settings of chronic inflammation are often pro-tumorigenic [151]. 

### 3.11. Tumor Cells

There is extensive evidence indicating that IFNIs act directly on premalignant and malignant cells through several mechanisms, including induction of cellular apoptosis and cell cycle arrest, to alter tumor growth and survival [47]. For example, IFNIs upregulate expression of the tumor suppressor p53, reducing cellular transformation [154]. Hematopoietic malignant cellular proliferation and survival is inhibited through IFNI-mediated activation of SIRT2/CDK9/STAT1 and ULK1 signaling [155,156]. Moreover, IFNIs exert an anti-tumor effect by upregulating NK ligands and MHC class I molecules on the tumor cell surface, increasing the immunogenicity of the tumor cells [89]. Several genotoxic anticancer therapies, such as radiation and chemotherapy, rely on the accumulation of DNA damage, DAMP production, and activation of IFNI-engaging pathways to promote an anticancer immune response [157,158]. However, activation of IFNI-associated pathways can also increase the expression of prosurvival ISGs, designated IFN-related DNA damage resistance signature (IRDS) genes, which can support tumor cell survival [159]. Moreover, in mouse mammary cancer cells, expression of an IFNAR1 mutant that is resistant to degradation did not alter the proliferation of these cells in vitro or when implanted subcutaneously in syngeneic mice but did increase tumor growth when implanted orthotopically into mammary glands [160]. These results highlight the importance of evaluating the role of IFN activity within a tumor’s niche and are consistent with the findings that increased expression of IFNAR1 is associated with a poor prognosis in breast cancer patients [160]. Similarly, investigations into mechanisms driving the malignant phenotype of glioblastoma have identified the IFNI-regulated human Schlafen *5* (*SLFN5*) gene as a critical driver of malignant characteristics [161]. These studies have shown that SLFN5 interacts with STAT1 and negatively controls its transcriptional activity. Thus, IFNIs can drive direct pro- and anti-tumor effects on tumor cells (Figure 1).

IFNγ also acts directly on tumor cells, regulating their survival and immunogenicity through multiple mechanisms [162,163]. For example, IFNγ increases the expression of MHC class I molecules on the surface of tumor cells, enhancing their immunogenicity and, consequently, making them more vulnerable to immune-mediated cell killing [162]. In addition to increased antigenicity, IFNγ induces tumor cell death using several direct mechanisms. IFNγ exposure inhibits tumor growth through upregulation of p21 and p27 and activation of p53-regulated signaling, leading to cell cycle arrest and apoptosis [109,164]. Moreover, IFNγ released by CD8+ T cells inhibits expression of SLC3A2 and SLC7A11 on tumor cells, leading to tumor ferroptosis [165]. IFNγ exposure can also result in autophagy-associated apoptosis of tumor cells via activation of cytosolic phospholipase A2-dependent production of mitochondrial reactive oxygen species [166]. Furthermore, in an in vivo model of pancreatic cancer, IFNγ treatment was shown to inhibit CXCL8 expression on tumor cells, reducing trafficking of suppressive CXCR2+CD68+ macrophages to the TME, restoring immune activity and response to anti-PD-1 therapy [167]. In another study, intratumoral NK-derived IFNγ was shown to induce expression of fibronectin 1 in tumor cells, changing the tumor architecture and reducing metastases formation in vivo [168].

In contrast, in an HBV-associated hepatocellular carcinoma (HCC) model, IFNγ produced by NK cells was found to induce STAT1-dependent expression of epithelial cell adhesion molecules, promoting the epithelial-to-mesenchymal transition of HBV surface antigen-positive hepatocytes and increasing HCC incidence in vivo [169]. In another study, NK-derived IFNγ-induced the expression of MHC class I molecules in leukemia cells and decreased their susceptibility to NK cytotoxicity [170], while in melanoma cells loss of IFNγ signaling components increased tumor cell sensitivity to NKs [171]. Using several mouse tumor models, IFNγ was shown to be essential for CTL-driven development of immune-resistant cancer cell clones through increased tumor cell genetic instability [172]. In another study, loss of Elf5-FBXW7 in triple negative breast cancer cells was shown to increase IFNγ signaling, leading to enhanced PD-L1 expression on tumor cells and accumulation of immunosuppressive neutrophils in the TME [173]. Moreover, sustained signaling through IFN-induced pathways has also been associated with resistance to immune checkpoint inhibitors (ICIs) through upregulation of PD-L1, PD-L2, CTLA-4, CIITA, IDO1, CXCL12, non-classical MHC antigens and nitric oxide production in tumor cells, which inhibit the ability of the immune system to recognize these cells [174,175,176,177,178,179,180,181,182]. In vivo studies of the diffusion of CD8+ T cell-derived IFNγ suggest that even few intratumoral CD8+ T cells secrete enough IFNγ to reach tumor cells > 800 μm away [183]. This may drive ongoing immunologic control of malignant cells, including variants that have lost antigen expression. However, this gradient may also lead to the upregulation of immunosuppressive proteins, such as PD-L1 and galectin-9, on tumor cells prior to T-cell arrival and immune recognition, creating immune resistant cancer cells [183]. Together, these observations highlight the dual direct role that IFNγ plays in malignant cells (Figure 2). 

As the field of oncology has increased its focus on personalized medicine, gene signatures and gene expression profiles (GEPs) have become important tools in understanding tumor biology [184]. Analyses of several tumors within T-cell inflamed TMEs identified a GEP that included IFNγ-inducible genes and could predict response to ICI therapy in melanoma and head and neck cancers [176] On the other hand, the IRDS is associated with resistance to radiation and chemotherapy [13,159]. In prostate cancer, an enhanced IRDS is associated with poor overall survival, and it was found to be more prevalent in African-American men than in European-American men [185]. These findings were correlated with the expression of a germline variant that regulates production of IFN lambda 4, a type III IFN commonly expressed in people of African ancestry [185]. African-American breast cancer patients also exhibit an elevated IFN signature compared to patients of European ancestry [186]. Further investigations into these gene signatures and associated changes within the anti-tumor immune response will likely contribute significantly to predict patient outcomes and response/resistance to chemo- or immunotherapies.

## 4. Resistance Mechanisms to Interferons 

IFN dysregulation can occur through several mechanisms including downregulation of signaling effector proteins (e.g., IFNAR, JAKs), loss or silencing of key signaling components (e.g., JAK1, STATs, IRFs), or through upregulation of negative regulators (e.g., SOCS1/3) [187,188]. Hypermethylation of the IFNγ promoter in CD8+ T cells isolated from cancer patients is associated with reduced IFNγ production following CD8+ T cell stimulation and with decreased T cell cytotoxicity [189]. Over time, tumor infiltrating T cells become less responsive to antigens, release less IFNγ, and kill fewer malignant cells. Several tumor types, including melanoma and head and neck cancers, are or become unresponsive to IFNs [190,191,192]. These escape mechanisms have been identified both in research models and in patient samples, suggesting they aid in the growth and survival of malignancies [161]. Moreover, TME stress factors, such as hypoxia [193], tumor-expressed inflammatory cytokines such as IL-1 [194] and tumor-derived extracellular vesicles [195], have all been shown to stimulate degradation and inactivation of IFNAR and suppress downstream signaling and induction of ISGs, blunting anti-tumor immune responses. Responses to anti-tumor treatments including chemotherapy, radiation and immunotherapy rely on IFN signaling and IFN-mediated immune responses [17,105,188]. Therefore, it is not surprising that loss of IFN signaling within the malignant and immune cells in the TME is associated with resistance to cancer treatments. Downregulation of IFNAR is found during tumor development in melanoma patients and expression of a non-degradable IFNAR1 mutant in mice was shown to delay the formation and progression of melanoma and increase responsiveness to BRAF or PD-1 inhibitors in vivo [196]. Further studies have also shown that loss of function mutations in the IFNR and alterations in the IFN signaling pathways allow immune escape from ICIs [36,197]. More specifically, loss of function mutations in JAK1/2, decreased phosphorylation of STAT1 and truncating mutations in the β2-microglobulin gene have all been identified in patients that develop resistance to ICIs [198,199]. Additionally, an acidic TME has been associated with decreased IFNγ production by NKs and enhanced tumor growth, suggesting that the TME is often not optimized to support IFNγ-mediated immune activity [200]. 

## 5. Conclusions

IFNs are essential components of immune cell activation and function, allowing elimination of malignant cells. However, due to feedback mechanisms developed to prevent over-inflammation and deleterious tissue destruction, IFN-activated signals can also suppress immune activity, possibly allowing tumor growth and escape from immunosurveillance [4,5,7,8]. These divergent responses depend on signaling duration, tumor characteristics, and the presence of other cytokines and immune cells within the TME. As research into potential therapeutic targets of components of IFN signaling and the immune response progresses, it will be important to take into account this dual function.
